# All-Optical Reversible Manipulation of Exciton and Trion Emissions in Monolayer WS_2_

**DOI:** 10.3390/nano10010023

**Published:** 2019-12-20

**Authors:** Chaoli Yang, Yan Gao, Chengbing Qin, Xilong Liang, Shuangping Han, Guofeng Zhang, Ruiyun Chen, Jianyong Hu, Liantuan Xiao, Suotang Jia

**Affiliations:** 1 State Key Laboratory of Quantum Optics and Quantum Optics Devices, Institute of Laser Spectroscopy, Shanxi University, Taiyuan 030006, Shanxi, China; chaoliyang1995@163.com (C.Y.); ago@sxu.edu.cn (Y.G.); liangxilongchn@163.com (X.L.); hansp1994@163.com (S.H.); guofeng.zhang@sxu.edu.cn (G.Z.); chenry@sxu.edu.cn (R.C.); jyhu@sxu.edu.cn (J.H.); jggp@sxu.edu.cn (S.J.); 2 Collaborative Innovation Center of Extreme Optics, Shanxi University, Taiyuan 030006, Shanxi, China; 3 Department of Physics, Shanxi Datong University, Datong 037009, Shanxi, China

**Keywords:** transition metal dichalcogenides, exciton, trion, reversible, all-optical

## Abstract

Monolayer transition metal dichalcogenides (TMDs) are direct gap semiconductors with promising applications in diverse optoelectronic devices. To improve devices’ performance, recent investigations have been systematically focused on the tuning of their optical properties. However, an all-optical approach with the reversible feature is still a challenge. Here we demonstrate the tunability of the photoluminescence (PL) properties of monolayer WS_2_ via laser irradiation. The broad-range and continuous modulation of PL intensity, as well as the conversion between neutral and charged excitons have been readily and reversibly achieved by only switching the two laser power densities. We attribute the reversible manipulation to the laser-assisted adsorption and desorption of gas molecules, which will deplete or release free electrons from the surface of WS_2_ and thus modify its PL properties. This all-optical manipulation, with advantages of reversibility, quantitative control, and high spatial resolution, suggests promising applications of TMDs monolayers in optoelectronic and nanophotonic applications, such as erasable optical data storage, micropatterning, and display.

## 1. Introduction

Atomically thin two-dimensional (2D) transition metal dichalcogenides (TMDs) with the chemical formula MX_2_ (M = Mo, W, and X = S, Se) have attracted great interest recently, due to their unique electric and optical properties as well as their potential applications in diverse optoelectronic devices [1,2]. Compared to their bulk crystals and the multilayer form, monolayer MX_2_ are direct gap semiconductors and manifest bright photoluminescence (PL) in the visible region even at room temperature [3]. Due to the spatial confinement of electron motion and reduced Coulomb screening in the 2D structures, monolayer MX_2_ are also emerging as a new platform for exploring many-body interactions, such as Auger recombination and exciton-exciton annihilation [4,5,6,7]. However, on the other hand, the large surface-to-volume ratio of monolayer MX_2_ makes them extremely sensitive to the changes of their surrounding environments, such as gating [8], doping [9], defects [10], temperature [11], excitation power [12] and collection locations [13]. Thus, exploring and manipulating the optical properties of these monolayer MX_2_ under different conditions are vital for the fundamental physics and optoelectronic applications.

Recently, unprecedented tunability of the optical properties of layered MX_2_ have been achieved both theoretically and experimentally. For example, by spinning coating *p*/*n*-type organic dopants (for instance, TCNQ and NADH molecules) or even biomolecules (such as DNA nucleobases) on monolayer MX_2_ [9,14,15,16], the conversion among neutral exciton and positive or negative trion can be undoubtedly determined, although these manipulations are irreversible. The reversible electrostatic tunability of the exciton charging effects in monolayer MX_2_ has been realized by using a field-effect transistor (FET) structure or molecular physisorption gating [8,17,18]. However, the contaminants and impurities are always inevitably introduced during the fabrication of the FET structure, which may significantly reduce the performance of optoelectronic devices. It can be partly avoided by using molecular physisorption gating, while the vacuum conditions and pumping apparatus impede their extensive applications [18]. Hence, an all-optical scheme that can readily operate in the atmosphere and reversibly manipulate the optical properties of monolayer MX_2_ without introducing extra impurities is highly desired.

Here, we show that the broad-range switch between exciton and trion of monolayer WS_2_, and thus the continuous modulation of its PL can be reversibly fulfilled by using two different laser power densities. Under the high power density, excitons will convert into trions, resulting in the quenching of PL intensity and redshift of its spectra. After quenching, the exciton can be recovered from trions by laser irradiation with the low power density. Consequently, the manipulation of excitons and trions, PL intensity, as well as PL spectra, can be reversibly manipulated by only switching the high and the low power densities of irradiation laser, which provides promising applications in erasable data storage.

## 2. Experimental Section

The optical experiments, including laser irradiation and PL collection, are performed by using a home-built scanning confocal microscope. The experimental schematic has been described in detail elsewhere [19,20], which can also be found in the Electronic Supplementary Information (ESI, Figure S1). Particularly, a 532 nm continuum-wave (CW) laser was used as both the irradiation source to modify the WS_2_ as well as the excitation laser to characterize its optical properties (PL imaging). The laser was focused by a dry objective (100×, NA = 0.9, NIKON Corporation, Tokyo, Japan) with the lateral dimension of the focal spot of about 1 μm. The WS_2_ sample was placed on a motorized three-dimensional piezoelectric translation nano-stage (PZT, Tritor, 200/20SG, Piezosystem jena, GmbH., Jena, Germany) with typical repeatability of less than 35 nm. The high spatial resolution guarantees that the laser-focused position on the WS_2_ sample during cycle irradiation is the same. PL imaging of monolayer WS_2_ was created by moving the sample with respect to the focused laser beam in a programmable and controlled way. PL intensity was collected by using the same objective. After passing through a dichroic mirror (Semrock, Di01-R532-25 × 36, IDEX Health & Science, LLC, Rochester, New York, USA) and a long-pass filter (Semrock, NF01-532U-25, IDEX Health & Science, LLC, Rochester, New York, USA) to block the back scatted laser as well as the background noise, PL was further filtered spatially using a 100 μm pinhole and then divided into two beams through a beam splitter. The PL intensity and its spectrum were synchronously recorded, by using a single-photon detector (SPCM-AQR-15, PerkinElmer Inc., Waltham, Massachusetts, USA) and a monochromator equipped with a cooled charge-coupled device (CCD, PIXIS, Princeton Instrument Inc., Trenton, NJ, USA), respectively. The incident laser power used in this experiment was ranging from 0.1 mW to 10 mW, calibrated using a power meter (Nova II, P/N7Z01550, Ophir Corporation, New Castle, Pennsylvania, USA) before each measurement.

## 3. Results and Discussion

### 3.1. Sample Characterizations

**Figure 1:**
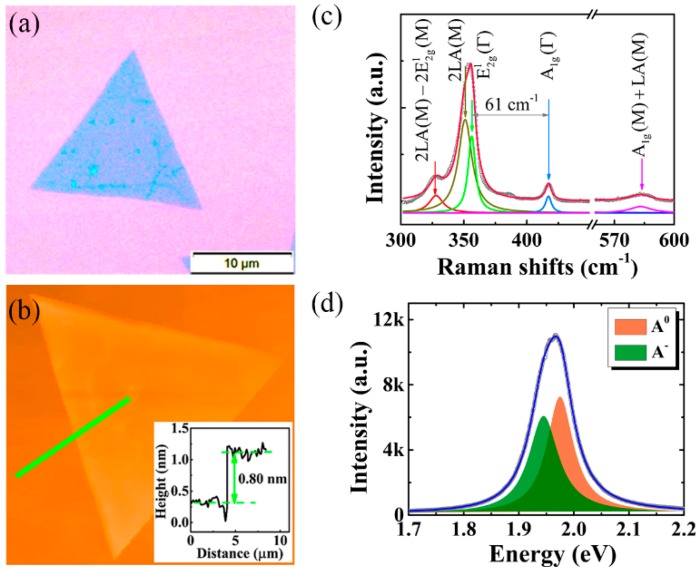
Characterizations of the prepared sample. Optical image (**a**) and atomic force microscopy (AFM) image (**b**) of WS_2_ prepared by chemical vapor deposition (CVD). The inset is the height profile of the selected line. (**c**) Raman spectroscopy of the prepared WS_2_ excited by 532 nm. The vibrational modes for prominent peaks have been assigned. (**d**) Photoluminescence (PL) spectra of monolayer WS_2_, which is fit to Lorentzians (orange is the exciton component, A^0^; green is the trion component, A^−^).

In the experiment, WS_2_, purchased from SixCarbon Technology (Shenzhen, China) was first grown on a sapphire substrate by using the conventional chemical vapor deposition (CVD) method. Considering that the switch between excitons and trions of monolayer MX_2_ on SiO_2_/Si substrate was more pronounced [21], we then transferred the WS_2_ to a SiO_2_/Si substrate with a poly (methyl methacrylate) (PMMA) assisted method [22]. Figure 1a presents an optical image of a typical sample. Isolated flakes with a triangular shape and edge lengths ranging from 10 to 20 μm can be clearly observed. The thickness of these WS_2_ flakes has been explored by atomic force microscopy (AFM) characterization, as shown in Figure 1b. The thickness of −0.8 nm (the cross section height is presented in the inset) confirms that the flake is a monolayer [23]. The relatively homogeneous color contrast in the AFM image also indicates that the basal plane of the monolayer WS_2_ is flat and uniform. We additionally note that the measured thickness is in agreement with the result of the Raman spectroscopy, as displayed in Figure 1c.

Raman spectroscopy was performed by a LabRAM HR Raman microscope (Horiba Inc., Kyoto, Japan) using the 532 nm laser excitation. The 521 cm^−1^ phonon mode from the Si substrate was used for calibration. As presented in Figure 1c, the peaks located at 417 cm^−1^ and 356 cm^−1^ can be attributed to the first order of the out-of-plane A_1g_(Γ) and in-plane E^1^_2g_(Γ) modes of WS_2_. A frequency difference of 61 cm^−1^ between the A_1g_(Γ) and E^1^_2g_(Γ) modes is definitely determined. These signatures agree well with previous Raman studies of monolayer WS_2_ [24,25]. Furthermore, the multi-peak Lorentzian fits shown in Figure 1c clearly reveals that some high order vibrational modes contribute to the Raman signal as well. As it is widely accepted, the broadening and strong intensity of E^1^_2g_(Γ) peak can be attributed to a second-order longitudinal acoustic phonon [26], 2LA(M) mode, emerging at 351 cm^−1^. An even weaker and lower peak (328 cm^−1^) has been attributed to the 2LA(M)-2E^1^_2g_(M) process. The high frequency mode beyond Si located at 583 cm^−1^ is generally attributed to A_1g_(M) + LA(M) [23,27]. Here, E^1^_2g_(M) and A_1g_(M) are the in-plane and out-of-plane vibrational modes related to the phonon dispersion near the M point of the Brillouin zone.

As expected, strong and uniform PL is observed at room temperature over the monolayer triangular shape, with the peak near 1.96 eV, as shown in Figure 1d. The peak can be well fitted by two Lorentzian functions. We attribute the higher peak at 1.98 eV to the neutral exciton emission (A^0^), and the lower peak at 1.94 eV to the negative charged exciton emission, known as trion (A^−^). The energy difference between the fitted two peaks is about 40 meV, reasonably coinciding with the trion dissociation energy reported [4,28], which is the sum of the trion binding energy and the Fermi level in the conduction band [4,7]. The slight discrepancies between reported works probably originate from the difference of two dimensional electron gas (2DEG) concentration in each WS_2_ sample, as discussed by Jadczak et al. [29]. Another possible reason is that the unequal excitation powers may lead to the renormalization of band structures [12]. Here, the trion is formed by an electron binding with an exciton, due to the charged feature of monolayer WS_2_ and the extra free electrons on its surface. The formation of trions will suppress the radiative recombination of neutral excitons and thus reduce PL intensity as well as change the emission energy. Therefore, the PL can be broadly manipulated by switching the ratio of excitons and trions. Hereinafter, we show that this manipulation can be readily and reversibly achieved by using laser irradiation with only two power densities (one high and one low), rather than a series of power densities, as reported in previous works [12,21,28].

### 3.2. All-Optical Reversible Manipulation of the Exciton and Trion Emissions

**Figure 2:**
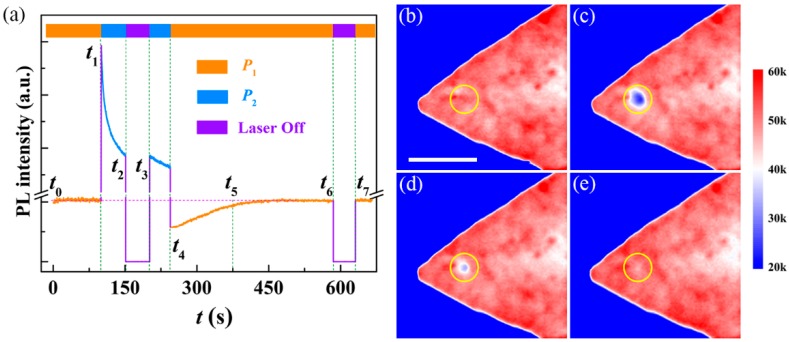
(**a**) PL trajectory of monolayer WS_2_ varied as laser irradiation with two power densities. In this set of experiments, *P*_1_ and *P*_2_ are 20 kW/cm^2^ and 900 kW/cm^2^, respectively. (**b**–**e**) PL images of monolayer WS_2_ under the laser excitation of *P*_1_ at time *t*_0_, *t*_4_, *t*_5_, and *t*_6_, respectively. Scale bar: 5 μm.

Figure 2a depicts PL intensity of monolayer WS_2_ varied as laser irradiation with the two power densities. At the initial stage with the low power excitation (*P*_1_ = 20 kW/cm^2^), the absence of changes in both PL intensity (*t*_0_ to *t*_1_ in the figure) and spectra (see Figure S2 in ESI) hints that the crystal structure and optical properties of monolayer WS_2_ are maintained during this stage. However, an unanticipated PL quenching emerged when the power density was switched to high (*P*_2_ = 900 kW/cm^2^). This quenching is a time-consuming process (typically more than 500 s to stabilize, see Figure S3 in ESI), rather than instantaneous. To understand this phenomenon, we switched off the laser for a duration (about 50 s from *t*_2_ to *t*_3_) during the quenching process and then switched on the laser again, as shown in Figure 2a. Comparing the PL intensity before laser off (*t*_2_) and after laser on (*t*_3_), we note that no significant changes occur. Thus, we suggest that the thermal effect, because of the strong excitation laser, can be excluded in this process. Due to the quenching under high power excitation, PL will become weaker compared with the initial stage, as shown in Figure 2b,c (see ESI for experimental details). We have confirmed that this PL medication can held after removing the irradiation laser under the ambient condition without any special protection (Figure S4). Amazingly, PL can be restored to the initial intensity by laser irradiation with an appropriate power density (as *P*_1_ used here and the details will be discussed below), as presented in Figure 2d,e, respectively. This restoration is robust and stable, as the *t*_6_ and *t*_7_ shown in Figure 2. We also confirmed that this recovery process could not be achieved under ambient atmosphere (Figure S5), unless irradiation with the optimized laser power density (as 20 kW/cm^2^ used here). Undoubtedly, an all-optical reversible manipulation on the PL intensity of monolayer WS_2_ has been fulfilled. Compared with previous works that modified PL of layered MX_2_ by thermal annealing [11,30], molecular doping [9,16], and physisorption gating [18], our all-optical manipulation not only holds the merits of reversibility but also maintains the high spatial resolution (limited by diffraction). In particular, this high spatial resolution provides promising applications in optical data storage, display technology, and relevant optoelectronic devices [31,32].

**Figure 3:**
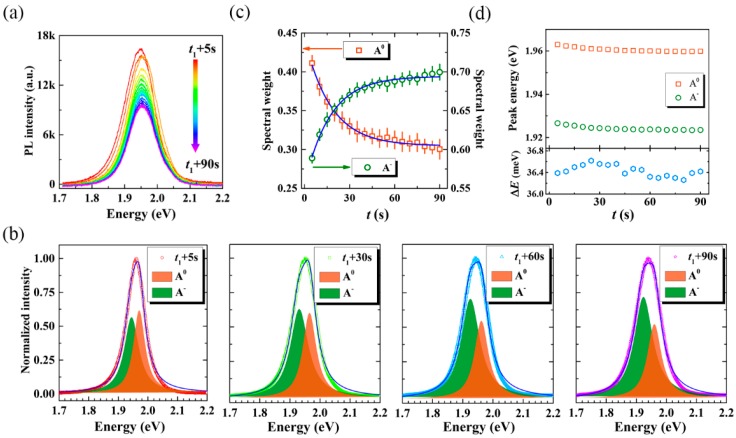
Evolution of PL spectra of monolayer WS_2_ obtained during the quenching process with the excitation of 532 nm and the power density of 900 kW/cm^2^. (**a**) PL spectra as a function of laser irradiation duration from *t*_1_ + 5 s to *t*_1_ + 90 s, as labeled in Figure 2a. (**b**) Representative PL spectra (normalized to the maximum PL intensity) under four different laser irradiation durations. All the spectral profiles are deconvoluted into two peaks (neutral exciton, A^0^, and trion, A^−^) using Lorentzian curves. (**c**) Spectral weights and (**d**) peak energies of A^0^ and A^−^ as a function of laser irradiation durations, respectively. The bottom panel presents the trion dissociation energy, Δ(*E_A_^0^* − *E_A_^−^*), varied as laser irradiation during the quenching processing.

To elucidate the underlying physical mechanisms relevant to the reversible manipulation, we conducted PL spectra during the quenching process under the high power excitation and the recovery process under the low power excitation, respectively. Figure 3a clearly demonstrates that the intensity of PL spectra decreases gradually as a function of laser irradiation duration. Meanwhile, the full width at half maximum (FWHM), and the peak position of the PL spectra also exhibit slight changes during the quenching process. To interpret these spectral changes, all the spectral profiles are decomposed into two Lorentz peaks. As presented in Figure 3b, with the increasing duration of laser irradiation, the relative proportion of A^0^ decreases gradually, while that of A^−^ increases. By dividing the integral intensity of each component to the full spectra, the spectral weights of both A^0^ and A^−^ can be determined, as shown in Figure 3c. Note that, the weight of A^0^ converts into A^−^ rapidly at the beginning of the quenching process, then slows down and tends to stabilize. The conversion rate can be determined empirically by fitting the two traces with a single exponential function.

(1) $$W_{A^{0}/A^{-}}(t)=W_{A^{0}/A^{-}}(0)+a_{A^{0}/A^{-}}\times \exp(k_{A^{0}/A^{-}}\cdot t)$$

Here, $W_{A^{0}/A^{-}}\left(t\right)$ is the spectral weight of A^0^ or A^−^ at time *t*, *k*_A_^0^(*k*_A_^−^) is the rate of A^0^ (A^−^) converting into A^−^ (A^0^). Based on the fitted curves (as the solid lines shown in Figure 3c), *k*_A_^0^ and *k*_A_^−^ are determined to be 5.8 × 10^−2^ s^−1^. Furthermore, we note that the peak position of A^0^ redshifts from 1.963 eV to 1.960 eV in this process, and that of A^−^ redshifts from 1.927 eV to 1.924 eV, respectively, as shown in Figure 3d. Nevertheless, their energy differences, Δ(*E*_A_^0^ − *E*_A_^−^), namely the trion dissociation energy, remain almost unchanged during this process and agree well with reported values of about 34 meV [33,34]. Thus, we can conclude that the quenching of PL is originating from the conversion from A^0^ to A^−^.

**Figure 4:**
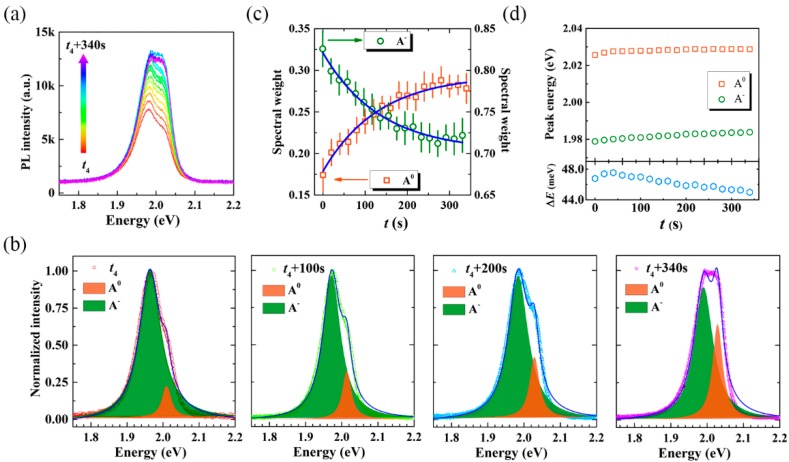
Time evolution of PL spectra obtained during the recovery process with the excitation of 532 nm and the power density of 20 kW/cm^2^. (**a**) PL spectra as a function of laser irradiation duration from *t*_4_ to *t*_4_ + 340 s. (**b**) Representative PL spectra (normalized to the maximum PL intensity) under four different laser irradiation durations. All the spectral profiles are deconvoluted into two peaks. (**c**) Spectral weights and (**d**) peak energies of A^0^ and A^−^ as a function of laser irradiation durations, respectively. The bottom panel presents the trion dissociation energy varied as laser irradiation during the recovery processing.

We also studied the time evolution of PL spectra in the recovery process after quenching, which confirms that the restoration of PL results from the conversion from A^−^ to A^0^, as presented in Figure 4. In this stage, PL spectra increase gradually, accompanied by characteristic changes of the emission profiles. Based on the Lorentzian fits shown in Figure 4b, the increase of A^0^ component can be clearly determined. One may find that a significant difference is present between the spectral profile for the end of the quenching process (i.e., the last spectrum shown in Figure 3b) and the beginning of the recovery process (i.e., the first spectrum shown in Figure 4b). This can be understood as the A^−^ component dominates the spectra at the excitation with the low power density. The spectral weights of A^0^ and A^−^ as a function of laser irradiation duration in this stage have been presented in Figure 4c, from which the conversion rates of *k’*_A_^0^ and *k’*_A_^−^ are determined to be 7.7 × 10^−3^ s^−1^ by Equation (1). That’s to say, the conversion rate from A^−^ to A^0^ in the recovery process is almost one order of magnitude slower than the conversion rate from A^0^ to A^−^ in the quenching process (5.8 × 10^−2^ s^−1^). As expected, the peak positions of both A^0^ and A^−^ are blue-shift in this stage. Intriguingly, the energy difference between exctions and trions reduces from 48 meV to 44 meV during the recovery process, as presented in Figure 4d. This result probably originates from the reduce of the 2DEG, which scales down the Fermi energy, as suggested by Jadczak [29]. Additionally, both the peak position and trion dissociation energy are higher than that in the quenching process with the high power excitation. It has been speculated that this is a result of band structure renormalization under the high excitation power [12].

**Figure 5:**
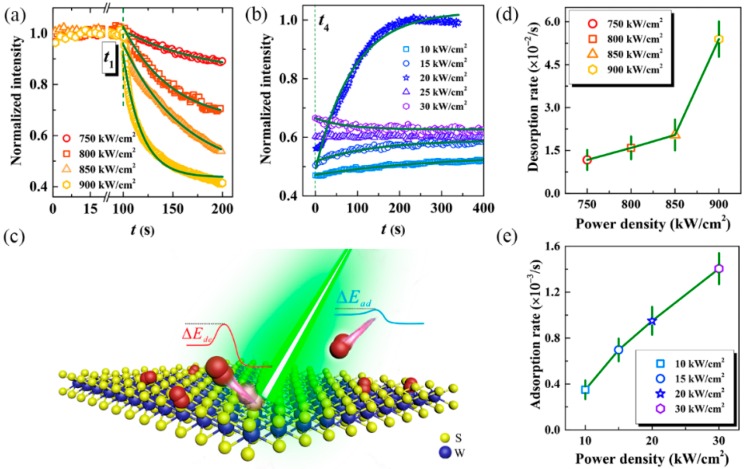
PL evolution and the proposed mechanism. Normalized PL trajectories of monolayer WS_2_ under different power densities in the quenching (**a**) and recovery (**b**) processes. Solid lines are the fitting results according to Equations (2) and (3), respectively. (**c**) Schematic of the proposed mechanism. Laser-induced heat effect will assist the adsorption and desorption of gas molecules from the surface of monolayer WS_2_, resulting in the depletion and release of free electrons. The energy barriers during the adsorption and desorption processes have been schematically illustrated. (**d**) and (**e**) are the fitted desorption and adsorption rates derived from the solid lines in (**a**) and (**b**), respectively.

To gain further insight into the nature of this effect, we performed this reversible manipulation under various laser power densities. As shown in Figure 5a, the quenching process becomes weaker as the irradiation power density decreases from 900 kW/cm^2^ to 750 kW/cm^2^. This process will be even absent when the power is lower than −400 kW/cm^2^. Conversely, the behaviors in the recovery process are more plentiful. In the relatively low irradiation power density (such as 10 kW/cm^2^ shown in Figure 5b), PL will be enhanced slightly but pretty slowly, rather than restored to the initial intensity. As the power density of irradiation laser increases, this situation will improve gradually and even reach the initial intensity under appropriate power density, such as 20 kW/cm^2^ shown in Figure 5b. However, PL may be maintained or even quenched mildly when the irradiation power density further increases, as 25 kW/cm^2^ and 30 kW/cm^2^ used in the experiments.

### 3.3. Proposed Mechanism to Explain the Manipulation

Here we propose a potential mechanism to explain the observed results. Laser irradiation with strong power density may result in the structural degradation of layered 2D materials and synchronously modify their optical properties, such as PL enhancement and energy shifts [12,35,36]. However, this kind of manipulation is destructive and non-reversible, and thus it can be excluded here. Previous works have also reported that PL quantum yield of monolayer MoS_2_ can be availably modulated by physical adsorption/desorption of O_2_ and/or H_2_O molecules through pumping up or down the gas pressure [18]. This reversible manipulation can provide orders of magnitude broader control of PL emission. Inspired by this result, we attribute our reversible manipulation to the laser-assisted adsorption/desorption of gas molecules (such as O_2_ and H_2_O) on the surface of monolayer WS_2_, as illustrated schematically in Figure 5c. Here we assume that (i) activation energy is required for both adsorption (Δ*E_ad_*) and desorption processes (Δ*E_de_*). Furthermore, the activation energy of the desorption process is higher than that of the adsorption process (Δ*E_de_* > Δ*E_ad_*). (ii) We also propose that laser irradiation will heat the layered samples and thus provide the activation energy to adsorb or desorb the gas molecules. Although activation energy is required, gas molecules will still accumulate on the surface of monolayer WS_2_ and reach saturation after exposing in the air for a long period (the sample has been exposed in air for months before performing this experiment), due to that the activation energy of desorption is higher than that of the adsorption process. Considering that WS_2_ monolayer maintains abundant free electrons on its surface [18], we can conclude that the adsorption of gas molecules will deplete the free electrons, while desorption will increase. Consequently, the amounts of excitons and trions can be switched by these two processes.

(I). At the initial stage, monolayer WS_2_ is irradiated by the low power density. Hence the laser-induced heat effect is not pronounced. Therefore, the activation energy is not high enough for desorbing gas molecules from the surface. Although adsorption may occur during this stage, the molecule concentration has already reached saturation before laser irradiation. Consequently, no more gas molecules can be adsorbed, and PL of monolayer WS_2_ will maintain, as *t*_0_ to *t*_1_ shown in Figure 2a.
(II). When the activation energy for desorbing gas molecules has been reached by laser irradiation with the high power density, the adsorbed gas molecules will fly away from the surface of monolayer WS_2_. Even though adsorption also occurs during this stage, we can still image that the number of desorbing molecules is much larger than that of adsorbing. This is readily understood by considering that the dense pre-adsorbed molecules can fly away directly and rapidly, while the new-adsorbed molecules have to fly from the ambient atmosphere. Consequently, the concentration of adsorbed molecules will decrease while the free electrons will increase, resulting in the conversion from A^0^ to A^−^ and the quenching of PL, as *t*_1_ to *t*_2_ shown in Figure 2a. The increased power density will improve the heat effect and thus increase the activation energy. According to the Arrhenius law, the desorption rate will increase synchronously. Thus, PL quenching will become more significantly under higher power density, as PL trajectories presented in Figure 5a.
(III). When the irradiation laser is tuned from the high power density to the low power density (such as *P*_2_ to *P*_1_ shown in Figure 2a), the desorption will be switched off while the adsorption may still survive. In the case, the molecule concentration adsorbed on the WS_2_ surface will increase, and thus the free electrons will be depleted. Consequently, the conversion from A^−^ to A^0^ happens and PL will be restored, as *t*_4_ to *t*_5_ shown in Figure 2a. Similarly, the recovery process becomes obvious as the power density increases. However, PL may stand at a plateau level or even quench again when the power density is high enough so that the desorption process is activated again, as 25 kW/cm^2^ and 30 kW/cm^2^ shown in Figure 5b.

By using the pseudo-first-order model to describe the adsorption/desorption process where adsorption/desorption rate is directly proportional to the difference between equilibrium and current molecule concentration over time, *N*(t) [37]. As a consequence, *N*(t) can be expressed as (see ESI for detailed derivation):

(2) $$N(t)=N(0)\times e^{-k_{de}(P_{Laser})\cdot t},$$

(3) $$N(t)=N(0)-(N(0)-N\prime (0))\times e^{-k_{ad}(P_{Laser})\cdot t},$$

Equation (2) describes the desorption process, and Equation (3) gives the adsorption process (the formula for the combination of the two processes has also been provided in the ESI), where *N*(0) is the saturation concentration, *N’*(0) is the initial concentration of the recovery process (i.e., the molecule concentration at *t*_4_ in Figure 2a). *k_de_*(*P_Laser_*) and *k_ad_*(*P_Laser_*) are the rate constants of desorption and adsorption at a certain power density, respectively. According to the Arrhenius law, the higher the power density (thus the more pronounced the laser-induced heat effect), the larger the rate constants. As the solid lines shown in Figure 5a,b, PL quenching and recovery processes can be well described by Equations (2) and (3), respectively. The determined rate constants have been presented in Figure 5d,e, respectively. As we expected, both desorption and adsorption rates increase as the power density increases. Furthermore, the desorption rate at the power density of 900 kW/cm^2^ is 5.4 × 10^−2^ s^−1^, which is in good agreement with 5.8 × 10^−2^ s^−1^, the conversion rate from A^0^ to A^−^ (as presented in Figure 3c). The adsorption rate at the power density of *P*_1_ also agrees reasonably well with the conversion rate from A^−^ to A^0^ (9.8 × 10^−3^ s^−1^ vs. 7.7 × 10^−3^ s^−1^). These results strongly support our assumption.

### 3.4. Discussion

Our adsorption and desorption model can be further verified by putting the sample under a blowing N_2_ atmosphere and a vacuum chamber. As expected, the reversible manipulation is fully lost in these two conditions (Figure S6). At first glance, our model has explained all the results successfully; however further experiments are still necessary to understand this reversible manipulation. Firstly, we attribute the PL quenching and recovery process to the physical desorption or adsorption of gas molecules (such as O_2_ and H_2_O) and use the activation energy to explain the results at different power densities. While physical adsorption is generally treated as a non-activated process with a rapid rate (even instantaneous), thus the origin of the activation energy assumed here is still elusive. Secondly, the exact values of activation energy for both adsorption and desorption are still unknown, although the activation energy of desorption higher than that of adsorption has been assumed in the context. These two values might be uncovered by further theoretical calculations and experimental designs, for example, performing this reversible manipulation under different temperature conditions. One may also note that a large redshifts (−50 meV) of exciton and trion emerge between the high (Figure 3d) and low (Figure 4d) power excitations. This large energy difference is probably originating from the local heating effect. The previous temperature-dependent PL measurements inspires us this redshift can be described by some semiempirical functions (such as Varshni’s equation) [8,11,38,39]. By determining the corresponding parameters via temperature-dependent PL spectra under extremely low power excitation, we can derive the temperature under different power densities directly. Combing with the corresponding adsorption and desorption rates, the activation energy can be demonstrated via Arrhenius law. However, the exact values require a further temperature-dependent study, which is currently underway. Thirdly, although Equations (2) and (3) can fit the results perfectly in most occasions, some of them still deviate from this model slightly, such as the quenching curve at the power density of 900 kW/cm^2^ and the recovery curve at the power density of 20 kW/cm^2^ shown in Figure 5a,b, respectively. The bi-exponential functions are more reasonable than Equations (2) and (3) (see Figure S7 in ESI for the detailed comparison). This deviation hints that more complicated reactions have occurred in this reversible manipulation. Finally, although promising applications have been proposed and reversible micropatterning on monolayer WS_2_ has been presented in principle (Figure 2d,e), some more practical and conceptual devices, especially erasable optical data storage, are still in design.

## 4. Conclusions

In conclusion, we have shown an all-optical manipulation on PL of monolayer WS_2_ that the conversion between excitons and trions can be readily and reversibly fulfilled by switching the power density of irradiation laser. The conversion rates between excitons and trions have been determined by analyzing PL intensity and spectral changes during the manipulation. A potential model, attributing the reversible manipulation to the laser-assisted adsorption and desorption of gas molecules that will deplete or release free electrons from the surface of WS_2_, has been proposed and explained the experimental results perfectly. The conversion rates between excitons and trions are in good agreement with the adsorption/desorption rates, further confirming our model. Our findings not only enable nondestructive, reversible, quantitative control of PL emission of monolayer WS_2_ without electrostatic gating but also provide an all-optical manipulation at desired locations on layered 2D materials with high spatial resolution. These features enable promising applications in micropatterning, erasable optical data storage, and display technology.

## Supplementary Materials

The following are available online at https://www.mdpi.com/2079-4991/10/1/23/s1, Figure S1: Schematic diagram of the experimental setup, Figure S2: PL evolution in the initial stage (from *t*_0_ to *t*_1_) with low power density (20 kW/cm^2^). Figure S3: PL trajectory of monolayer WS_2_ during the quenching process. The excitation power density is 900 kW/cm^2^. Figure S4,S5: Stability of PL modification under ambient atmosphere. Figure S6: PL behaviors under N2 and vacuum conditions. Figure S7: Simulating the quenching process at the power density of 900 kW/cm^2^ by different models. The derivation of the adsorption/desorption rate has also supplied.
