# Supplementary material for: All-Optical Reversible Manipulation of Exciton and Trion Emissions in Monolayer WS2
Source: Nanomaterials (Basel). 2019 Dec 20;10(1):23. doi: 10.3390/nano10010023 (PMC7023460; doi:10.3390/nano10010023)
Supplement: Supplementary file 1 [file nanomaterials-10-00023-s001.pdf]

Electronic Supplementary Information for

All-optical reversible manipulation of exciton and trion emissions in monolayer WS_2

Chaoli Yang,[1,3] Yan Gao,[1,2] Chengbing Qin,[1,3*] Xilong Liang,[1,3] Shuangping Han,[1,3] Guofeng Zhang,[1,3] Ruiyun Chen,[1,3] Jianyong Hu,[1,3] Liantuan Xiao,[1,3*] and Suotang Jia[1,3]

[1] State Key Laboratory of Quantum Optics and Quantum Optics Devices, Institute of Laser Spectroscopy, Shanxi University, Taiyuan, Shanxi 030006, China.

[2] Department of Physics, Shanxi Datong University, Datong 037009, Datong, China

[3] Collaborative Innovation Center of Extreme Optics, Shanxi University, Taiyuan, Shanxi 030006, China.

1. Schematic diagram of experimental setup

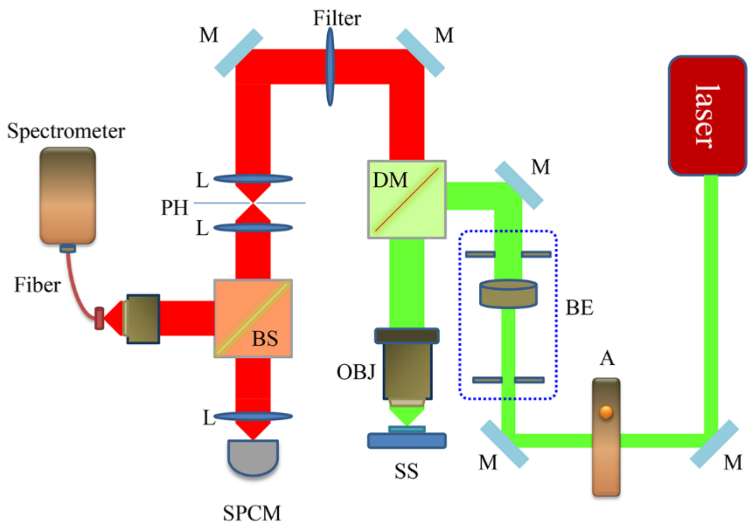

Fig. S1 Schematic diagram of experimental setup. M: Mirror; A: Attenuator; BE: Beam Expander; DM: Dichroic Mirror; OBJ: Objective; SS: Sample Stage; L: lens; PH: Pinhole; BS: Beam Splitter; SPCM: Single Photon Counting Modular.

2. PL evolution at the initial stage

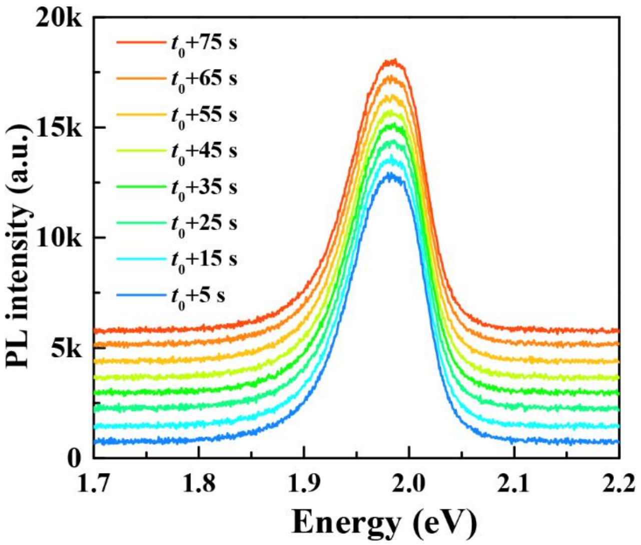

Fig. S2 PL evolution in the initial stage (from t_0 to t_1) with low power density (20 kW/cm^2). No significant difference can be found during this stage.

3. PL trajectory during the quenching process

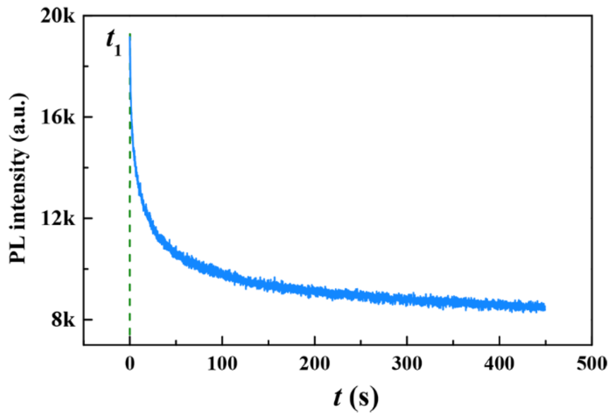

Fig. S3 PL trajectory of monolayer WS_2 during the quenching process. The excitation power density is 900 kW/cm^2.

4. Details about PL trajectory and PL imaging of Figure 2

The PL trajectory shown in Figure 2a was obtained step by step, rather than a continuous measurement. The experiments were performed as follows:

1) In the first step, we scanned the WS_2 sample voxel-by-voxel with a large area (typical 90 μm × 90 μm) by a low excitation power density (typical 20 kW/cm^2). The integration time for each voxel was 10 ms. Considering the low excitation power density and the short excitation time, the change of PL properties can be ignored.
2) Secondly, we selected an interested triangle WS_2 monolayer (typical 20 μm × 20 μm) and performed the scanning again to achieve PL image, as shown in Figure 2b.
3) Then, the laser was focused on a selected position. During the low irradiation power (20 kW/cm^2 in the manuscript), PL of this position has no significant change, as t_0 to t_1 (0 s to 100 s) shown in Figure 2a.
4) Later, we switched the irradiation laser from the low power density to the high power density (900 kW/cm^2) and monitored the PL behaviors. During this stage, we switched off the laser at t_2 (150 s) and switched on the laser again at t_3 (200 s).
5) In the fourth step, we switched off the high power density laser at t_4 (244 s) and performed PL imaging. Typically, PL image consists of an array of 100×100. Thus the scanning time for the full image was about 100 s, considering that the integration time for each voxel was 10 ms.
6) After PL imaging, we re-focused the selected position and recovered the PL intensity by using the low power density (20 kW/cm^2). PL images can be obtained at any time of the full process, such as t_5 and t_6, as performed in step 5.

Comparing with the large time scale for the recovering process (~500 s), the influence on the PL properties arising from the short scanning time (10 ms) can be ignored.

5. Stability of the PL modification under ambient atmosphere

The stability of PL modification has been confirmed by performing PL imaging of the irradiated locations at different times, as shown in Figure S4. The sample was first modified at 2018.11.1, 9:35 pm. Then we held the sample and experimental setup for overnight and performed PL imaging again at 2018.11.2, 9:58 am. No significant changes can be found between the two images. Further confirmation was taken at 2018.11.4, at 3 pm. Both the sample and optical setup were placed in the ambient condition without any protection. These results illustrate the robust of our approach. To get insight into the changes among PL imaging at different times, we plotted the PL intensities along the marked green lines, as presented in Figure S4d. The slight difference between three-time measurements probably originated from the slight alteration in laser powers. To eliminate this effect, we normalized the PL intensities, as presented in Figure S4e. The consistency for the three measurements proved that the modified PL intensity could be held after switching off the laser irradiation.

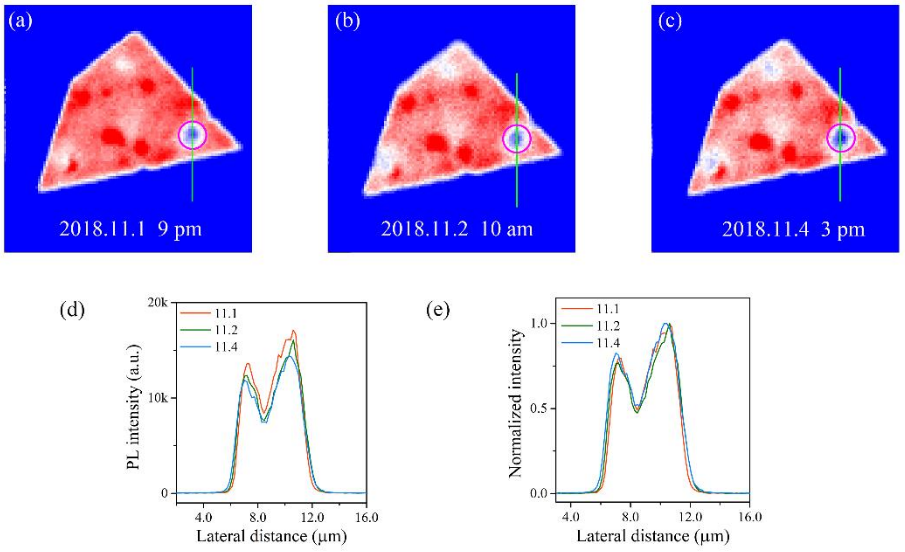

Fig. S4 (a)-(c) PL imaging of the modified monolayer WS_2 at different times. The

irradiated locations have been marked by the solid circles. (d) The PL intensities along the lines highlighted in a-c. (e) The corresponding normalized PL intensities.

We farther approved the robust of the PL modification by monitoring PL intensity against laser switching, as shown in Figure S5. We can find that the interrupted PL recovery process could be restarted after switching on the laser again. During laser off, the sample was stored in the ambient atmosphere without any further protection. No significant changes in the PL intensity can be determined.

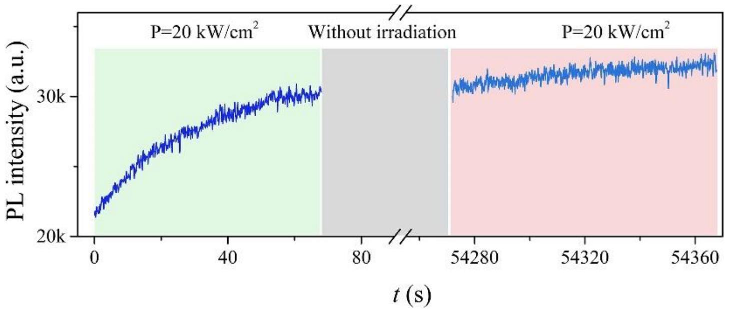

Fig. S5 Shutting down and bringing up the PL recovery process by switching off the irradiation laser.

6. Derivation of the adsorption/desorption rate

In the adsorption process, the equilibrium concentration of adsorbed molecules can be regarded as a constant, N_0 (in cm^{-2}), where the adsorption reaches saturation. Under the assumption of pseudo-first order model, the change of molecule concentration over time, N(t), is proportional to the difference the difference between equilibrium and current concentration, which can be expressed as:

$$\frac{dN(t)}{dt} = k_{ad}(P_{Laser}) \times (N(0) - N(t)) \qquad \text{(S1)}$$

where $k_{ad}(P_{Laser})$ is the rate constant of adsorption in s^{-1} at a certain laser power. The value of $k_{ad}(P_{Laser})$ depends on the laser power used to irradiate the monolayer WS_2, when the power is too low, the activation energy cannot overcome the barrier of the adsorption process, $k_{ad}(P_{Laser})$ is close to zero. According to the Arrhenius law, $k = Ae^{-E_a/RT}$, the high the irradiation power (and thus the pronounced the laser-induced heat effect), the large the $k_{ad}(P_{Laser})$. The integral form of equation S1 can be expressed as:

$$N(t) = N(0) - (N(0) - N'(0))e^{-k_{ad}(P_{Laser})\cdot t} = N(0) - \Delta N \times e^{-k_{ad}(P_{Laser})\cdot t} \qquad \text{(S2)}$$

where $N'(0)$ is the initial concentration of the recovery process (*i.e.* the molecule concentration at t_4 in Figure 2a).

On the other hand, the equilibrium condition of desorption process can be regarded as that all the adsorbed molecules have been lifted from the surface. That's to say, the equilibrium concentration of desorption process is zero. Similar to equation S1, the change of molecule concentration in the desorption process can be described as:

$$\frac{dN(t)}{dt} = k_{de}(P_{Laser}) \times (0 - N(t)) = -k_{de}(P_{Laser}) \times N(t) \qquad \text{(S3)}$$

where $k_{de}(P_{Laser})$ is the rate constant of desorption in s^{-1} at a certain laser power. Its

integral form can be given as:

$$N(t) = N(0) \times e^{-k_{de}(P_{Laser}) \cdot t} \tag{S4}$$

When the adsorption and desorption processes are both existing at the same time, the change of molecule concentration can be given by combining equation S1 and S3, as follows:

$$\begin{aligned} \frac{dN(t)}{dt} &= -k_{de}(P_{Laser}) \times N(t) + k_{ad}(P_{Laser}) \times (N(0) - N(t)) \\ &= k_{ad}(P_{Laser}) \times N(0) - (k_{ad}(P_{Laser}) + k_{de}(P_{Laser})) \times N(t) \end{aligned} \tag{S5}$$

The formula of the combining process can be expressed as:

$$\begin{aligned} N(t) = &\frac{k_{ad}(P_{Laser}) \times N(0)}{k_{ad}(P_{Laser}) + k_{de}(P_{Laser})} \\ &- \frac{k_{ad}(P_{Laser}) \times N(0) - k_{ad}(P_{Laser}) + k_{de}(P_{Laser}) \times N'(0)}{k_{ad}(P_{Laser}) + k_{de}(P_{Laser})} e^{-k_{ad}(P_{Laser}) + k_{de}(P_{Laser}) \cdot t} \end{aligned} \tag{S6}$$

By assuming the absent of desorption process (*i.e.* $k_{de}(P_{Laser})$ equals to zore), we can simply obtain equation S2 from S6. Similarly, when the adsorption process is absent (*i.e.* $k_{ad}(P_{Laser})$ equals to zore), and the initial concentration equals to the equilibrium concentration, $N(0)$, the formula of S4 can be readily determined as well.

7. PL behaviors under N_2 and vacuum conditions

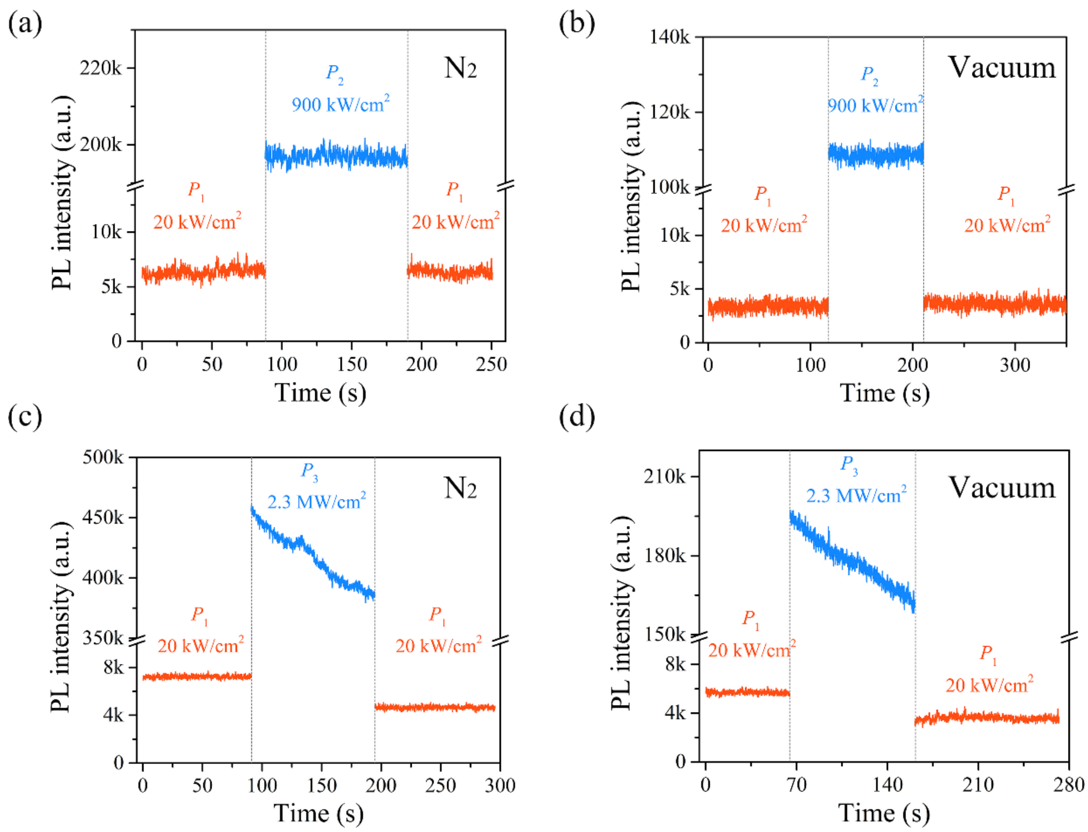

Fig. S6 PL behaviors of monolayer WS_2 under N_2 and vacuum conditions with different power densities. (a) With the typical low power density (20 kW/cm^2) and high power density (900 kW/cm^2), no obvious PL quenching or recovering can be found under both N_2 and vacuum conditions. Note that PL intensity under vacuum is slightly weak than that under N_2 atmosphere, probably due to that the molecules adsorbed on the defects under vacuum condition were less than that under N_2 atmosphere. (b) With higher power density (~2.3 MW/cm^2), PL shows slightly quenching under both conditions. This phenomenon can be attributed to the thermal degradation due to the extremely strong excitation laser, coinciding with previous works. However, these quenching PL cannot be reversed by laser irradiation with any low power densities.

8. Comparison the fitting results between single- and bi-exponential functions

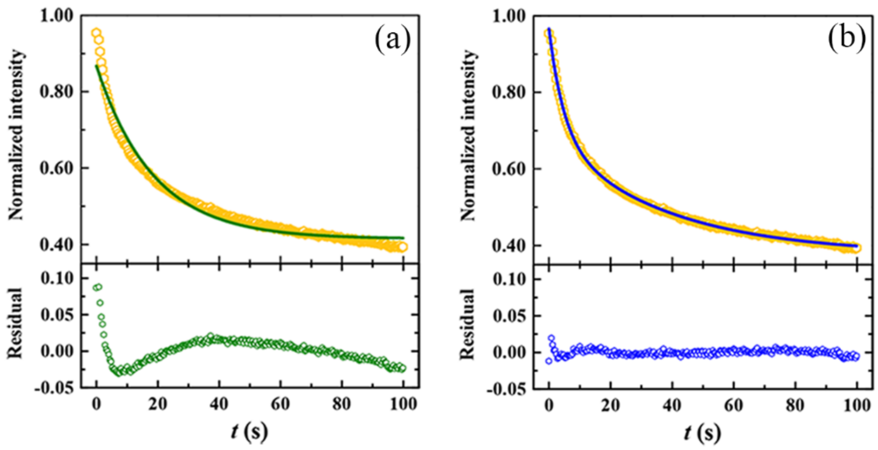

Fig. S7 Simulating the quenching process at the power density of 900 kW/cm^2 by different models. (a) The solid line in the upper panel is the single exponential fitting curve, the bottle panel is the fitting residual. (b) The solid line in the upper panel is the bi-exponential fitting curve, the bottle panel is the fitting residual. We can find that the bi-exponential function is more reasonable than single exponential function.
